# Analgesic efficacy of different volumes in erector spinae plane block in patients undergoing single level lumbar spine fixation: a non-inferiority randomized trial

**DOI:** 10.1186/s12871-025-03247-z

**Published:** 2025-09-01

**Authors:** Mohammad Fouad Algyar, Ahmed Anas Zahra, Ahmed Samir Elshikhali, Essam Ahmed Abdelhameed, Dalia Ahmed El Hefny, Saad Ahmed Moharam, Mohammed Said ElSharkawy, Omar Sayed Farghaly, Mohammed Awad Ahmed

**Affiliations:** 1 Anaesthesiology, Surgical Intensive Care and Pain Medicine, Faculty of Medicine, Kafrelsheikh University, Kafrelsheikh, Egypt; 2 Neurosurgery, Faculty of Medicine, Kafrelsheikh University, Kafrelsheikh, Egypt; 3 Neurosurgery, Faculty of Medicine, Tanta University, Tanta, Egypt; 4 Anaesthesiology, Surgical Intensive Care and Pain Medicine, Faculty of Medicine, Tanta University, Tanta , Egypt; 5 Anaesthesiology, Surgical Intensive Care and Pain Medicine, Faculty ofMedicine, AL Fayoum University, AL Fayoum, Egypt

**Keywords:** Analgesia, Erector spinae block, Lumbar spine fixation, Ultrasound, Non-inferiority trial, Postoperative pain

## Abstract

**Background:**

Using a lower volume (LVs) of local anaesthetic (LA) reduces the risk of toxicity, side effects, and cost. Our study assessed whether the lower LA volumes (10 or 15 ml) have the same analgesic efficacy as 20 ml of erector spinae plane block (ESPB) in single-level lumbar spine fixation.

**Methods:**

Our non-inferiority, randomised, double-blind trial recruited sixty cases scheduled for single-level lumbar spine fixation. All cases had bilateral ultrasound-guided (USG) ESPB before the surgery by bupivacaine 0.25% and were randomised into three groups according to the volume used: 10 ml for the E10 group, 15 ml for the E15 group, and 20 ml for the E20 group. The primary outcome was total morphine consumption. The secondary outcomes were pain scores, time of first analgesic, side effects, and patient satisfaction.

**Results:**

There was a non-inferior positive analgesic effect in terms of intraoperative fentanyl consumption, time till first rescue analgesia (*P* = 0.862), postoperative morphine within the first 24 and 48 h, and pain score in groups E10 and E15 compared to group E20 (*P* > 0.05). Patients who required intraoperative fentanyl were 4 (20%) in group E10, 2 (10%) in group E15, and 1 (5%) in group E20 (*P* = 0.322). Postoperative morphine within the first 24 h was 3.6 ± 1.23 mg in group E10, 3.3 ± 0.92 mg in group E15, 3.3 ± 0.92 mg in group E20 (*P* = 0.575), and at 48 h was 6.8 ± 1.65 mg in group E10, 6.2 ± 1.81 mg in group E15, 5.6 ± 1.76 mg in group E20 (*P* = 0.103). Intraoperative hemodynamic measurements, ambulation time, patient satisfaction, and complications were comparable among the three groups (*P* > 0.05).

**Conclusions:**

Preoperative ESPB is an effective analgesic technique for single-level lumbar spine fixation, with LVs proving non-inferior to higher volumes in clinical outcomes while potentially minimizing toxicity and side effects.

**Trial registration:**

Registration at clinical trial gov. (ID: NCT05892887). The date of the first registration submission was (2023-05-10), and the study started on 2023-06-01.

## Introduction

Globally, a dramatic rise in spine surgeries has been documented, making it one of the fastest-growing surgeries [1, 2]. Challenges with postoperative pain control are common with spine surgeries [3]. Delays in mobility, pulmonary and thromboembolic consequences, prolonged stays at hospitals, as well as chronic pain syndromes are all possible outcomes of inadequate pain management [4]. Therefore, clinical practice guidelines have emphasised using a multimodal analgesic strategy to improve recovery and pain management and enhance patient outcomes following spinal surgery [5–7].

Opioid-based analgesia has several adverse events (nausea, vomiting, respiratory depression, longer stays at the hospital, and substantial expenses), particularly for those administering high dosages. Therefore, finding alternative ways to decrease opioid consumption is essential for the postoperative period following lumbar spine surgeries [8].

The ESPB was initially identified by Forero et al. [9] as an interfacial plane block for an effective therapy for thoracic neuropathic pain. Several studies revealed that ESPB has prolonged postoperative pain relief, thereby decreasing the necessity for opioid consumption and its associated complications in lumbar spine surgery and lumbar vertebral surgical fixation procedures [10–14].

Previous studies used ESPB with a 20- or 30-ml volume of local anaesthetic (LA) in lumbar spine surgeries [10, 15, 16]. Studies about using LVs, such as 10- or 15-ml LA, are limited. We hypothesised that 10 or 15 ml LA volumes are non-inferior to 20 ml LA regarding analgesic efficacy. This led us to use LVs of LA to minimise the possibility of toxicity and side effects, such as seizures, cardiac arrest, or allergic reactions, and lower the cost.

The comparative effectiveness of analgesic therapies (LVs using ESPB for individuals undergoing single-level lumbar spine fixation) was the focus of this clinical experiment.

## Methods

### Ethics statement

The research was conducted from February 2023 to July 2023, following the approval of the Faculty of Medicine ethical committee, Kafr-Elsheikh University, Egypt, and registration of clinicaltrials.gov (ID: NCT05892887, the date of the first registration submission was 10/5/2023, and the study started on 1/6/2023). The trial was conducted following the Helsinki Declaration. Written consent was obtained from the patient.

### Study design

The non-inferiority, randomised, double-blind study involved sixty individuals aged between eighteen and sixty-five, the two sexes, physical status I-II according to the American Society of Anaesthesiology (ASA), scheduled for single-level lumbar spine fixation.

We excluded pregnancy, body mass index (BMI) above thirty kg/m^2^, kidney, liver, lung, or heart disorders, communication difficulties, contraindications to ESPB (such as bleeding disorder, anticoagulants, local infection, known allergies to LA), cases who had repeated back surgery, history of any specific substance abuse, and any psychiatric problem.

### Randomisation and blindness

Parallel to a computer-generated sequence, randomisation (a research randomiser website (randomizer.org)) was implemented through sealed opaque envelopes. Patients were administered preoperative bilateral USG-ESPB by bupivacaine 0.25% and were randomly assigned to three equal categories as follows: Group E10 received 10 ml on each side, Group E15 received 15 ml on each side, and Group E20 received 20 ml on each side.

Blinding of participants and evaluators was implemented, and an uninvolved pharmacist executed the bupivacaine solution’s preparation. All blocks were done utilising USG before general anaesthesia (GA) induction by one anaesthesiologist who did not participate in data collection or analysis.

All participants underwent comprehensive medical history, general examination, and laboratory testing. Patients were instructed about the Numerical rating scale (NRS), 0 to 10. Zero indicates the absence of pain, while ten indicates severe pain.

Following cannula insertion, all participants receive a premedication of midazolam at a dosage of two mg IV. They underwent monitoring utilising pulse oximetry, electrocardiogram, non-invasive blood pressure, capnography, and temperature probe.

### The USG-ESPB

ESPB was performed in the operating theatre before the induction of GA using Philips^©^ CX50 Extreme Edition. A curved probe, operating within the 2–5 MHz frequency range, was sagittally aligned adjacent to the targeted vertebral level while the subject rested in a prone position. The probe was shifted laterally by approximately three centimetres from the spinous process. Subsequently, the ES and transverse muscles were identified. Insertion of the needle occurred at the interface separating the ES muscle from the subjacent transverse process. Administration of two millilitres of saline ensued, verifying the plane of the ES muscle. Confirmation of the needle tip’s placement was achieved by observing the fluid dispersion, which elevated the ES muscle away from the transverse process shadow. The bilateral ESPB was established by administering 10, 15, or 20mL bupivacaine 0.25% into both sides according to the assigned group.

The block’s effectiveness was established by the lack of puncture at the injection site (T5-T6) thirty minutes after injection before the injection of GA.

For GA induction, propofol 1.5–2.5 mg/kg intravenous (IV) and fentanyl one µg/kg IV were each employed. To evaluate endotracheal intubation, cis-atracurium 0.15 mg/kg IV was administered. Anaesthesia maintenance was performed with 1-1.5% isoflurane in 50% oxygen and IV administration of maintenance doses of 0.03 mg/kg cis-atracurium.

Individuals underwent mechanical ventilation to maintain end-tidal CO_2_ 30–35 mmHg (inspiratory-to-expiratory ratio 1: 2, positive end-expiratory pressure 5 cm H_2_O, tidal volume 6–8 ml/kg, respiratory rate 10–14 cycles per minute). Extra fentanyl bolus dosages of one µg/kg IV were administered when heart rate (HR) increased or mean arterial blood pressure (MAP) was above 20% of the baseline. We used the bispectral index and a train of four monitoring to exclude insufficient anaesthesia or relaxation. The same surgical team performed all surgical procedures. HR and MAP were recorded at baseline, then every 15 min until the surgical procedure was completed.

Finally, all anaesthetics ceased, and the neuromuscular blockade was counteracted by neostigmine administration (0.08 mg/kg) and atropine (0.02 mg/kg), and then extubation was performed. Individuals were then sent to the post-anaesthesia care unit (PACU). When the modified Aldrete score exceeded 9, the patients were discharged from the PACU.

Pain (measured with NRS) was assessed in PACU 1,2, 4, 6, 8, 12, 18, 24, 36, and 48 h postoperative. Patients were given IV paracetamol 1 gm/8 h. If NRS > 4 was documented, rescue analgesia (morphine 3 mg IV) administration was done.

Time till the first rescue analgesia, the total amount of morphine in the 24th hour and 48th hour postoperative, and ambulation time were documented.

The patient satisfaction level was evaluated utilising a 5-point scale: (zero indicates extremely dissatisfied, one indicates unsatisfied, two indicates neither satisfied nor unsatisfied, three indicates satisfied), while four indicates extremely satisfied). The adverse events were documented, such as bradycardia (HR < 60 beats/min and was managed by atropine 0.6 mg IV), hypotension (MAP < 20% of baseline readings and was managed by ephedrine 5 mg IV and/or normal saline IVI), and postoperative nausea and vomiting (PONV) treated by ondansetron 0.1 mg/kg IV.

The primary outcome was the total morphine consumption during the initial twenty-four hours after surgery. The secondary one involved postoperative pain scores, time of first analgesic, side effects, and patient satisfaction after surgery.

### Sample size calculation

The PASS software (version 11; NCSS PASS, UT, USA) was employed to determine the sample size. The trial’s primary outcome involved total postoperative morphine administration. The sample size depended on these considerations: 95% confidence limit, 80% power of the study, group ratio 1:1, the standard deviation of the cumulative 24 h opioid administration was 6.81 mg with 20 ml ESPB according to a previous study [17], the non-inferiority margin was set to 6 mg, and four individuals were incorporated into both groups for dropout compensation and technique failure rate. Therefore, 20 patients were recruited in each group.

### Statistical analysis

SPSS v27 (IBM, Chicago, IL, USA) was used for statistical analysis. Histograms and the Shapiro-Wilks test were employed to normalise the data distribution assessment. The ANOVA (F) test was employed to analyse parametric quantitative data presented as mean and standard deviation (SD). The Kruskal-Wallis’s test was employed to analyse quantitative nonparametric data, which was presented as the median and IQR. The Chi-square test provided qualitative variables such as frequency and percentage (%). Statistical significance is determined by a two-tailed P value less than 0.05.

## Results

**Fig. 1:**
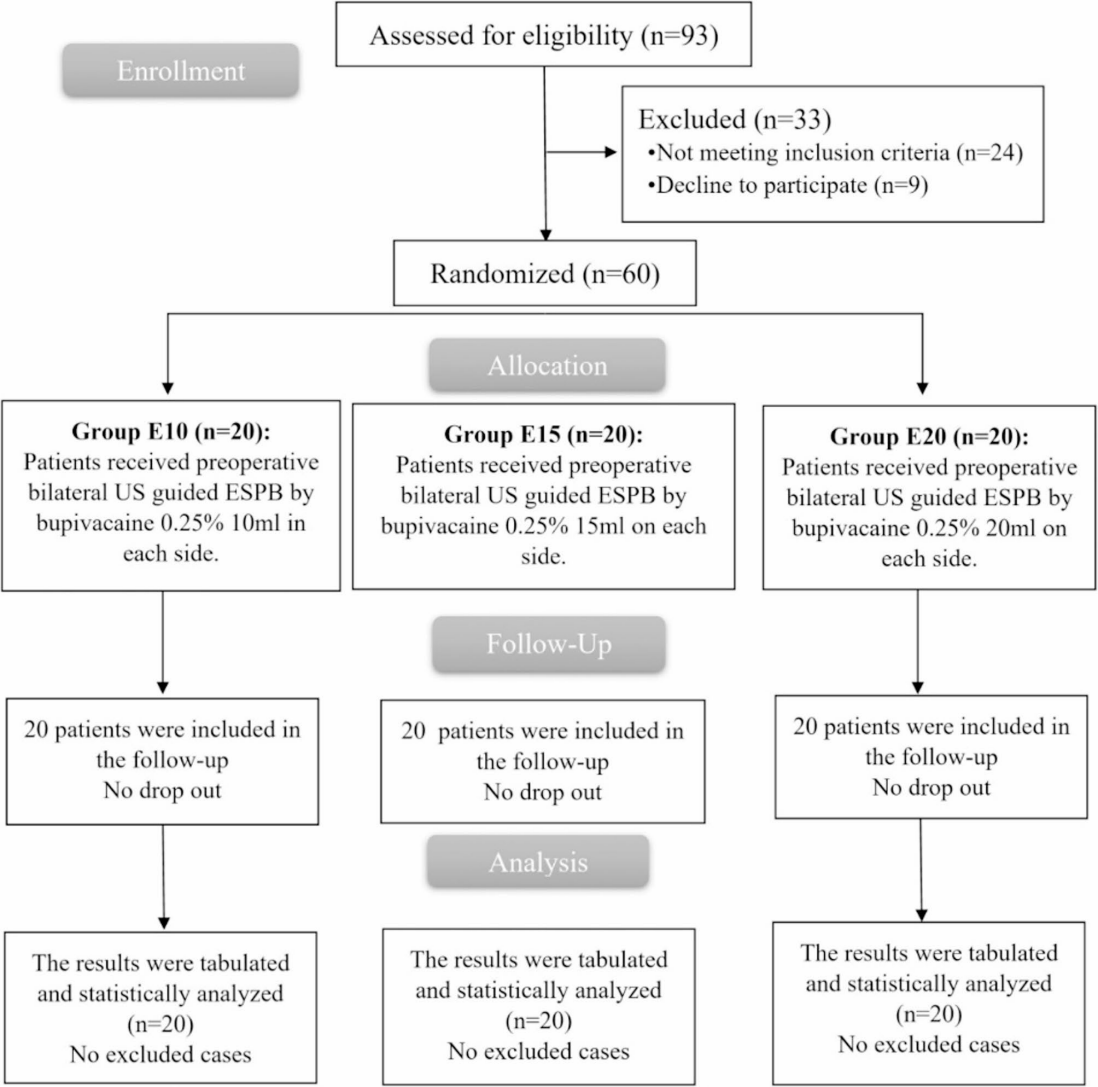
CONSORT flowchart of the enrolled patients

Our study involved 93 individuals evaluated for eligibility; 24 were excluded, and nine did not agree to participate. The remaining individuals were randomly selected and categorised into three equal categories (twenty participants within each). All of them underwent a follow-up as well as analysed statistically. Figure 1.

Demographic data and surgery duration were insignificantly varied among the three groups (*P* > 0.05). Table 1.

**Table 1:** Demographic data and duration of surgery of the studied groups

		Group E10 (*n* = 20)	Group E15 (*n* = 20)	Group E20 (*n* = 20)	*P* value
Age (years)		47.7 ± 13.07	46.3 ± 10.14	40.8 ± 11.64	0.151
Sex	Male	13 (65%)	12 (60%)	9 (45%)	0.414
	Female	7 (35%)	8 (40%)	11 (55%)	
Weight (kg)		74.9 ± 8.13	76.1 ± 11.31	73.2 ± 9.21	0.646
Height (cm)		176.4 ± 7.35	174.1 ± 7.62	172 ± 9.26	0.239
Body Mass Index (kg/m^2^)		24.2 ± 3.36	25 ± 2.92	24.9 ± 3.83	0.684
ASA physical status	I	14 (70%)	13 (65%)	15 (75%)	0.788
	II	6 (30%)	7 (35%)	5 (25%)	
Duration of surgery (min)		110.8 ± 26.87	99.3 ± 25.51	96.3 ± 23.89	0.173

Data are presented as mean ± SD or frequency (%). *ASA* American Society of Anaesthesiologists

**Fig. 2:**
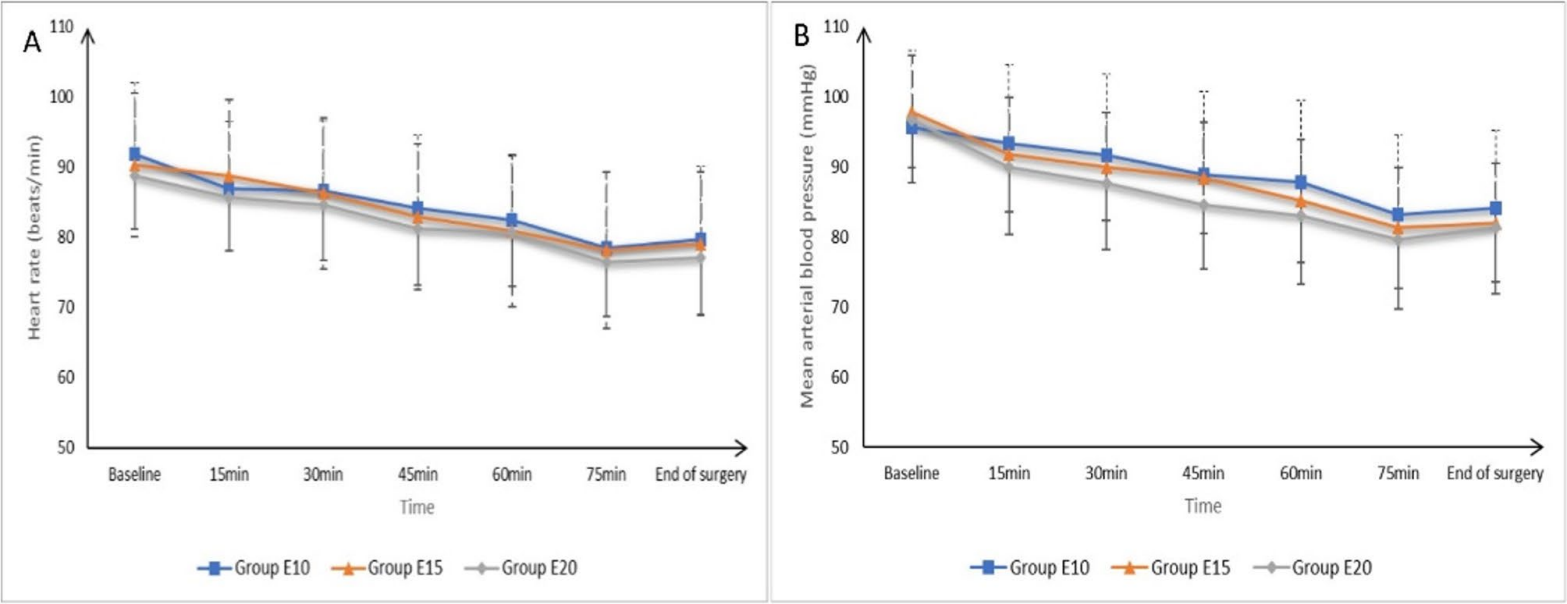
Intraoperative (**A**) heart rate, (**B**) mean arterial pressure measurements of the studied groups. Data are presented as mean ± SD

Baseline and intraoperative HR and MAP measurements were not significantly different among all categories (*P* > 0.5). Figure 2.

NRS measurements at PACU, 1, 2, 4, 6, 8, 12, 18, 24, 36, and 48 h were insignificantly varied among all groups (*P* > 0.05). Table 2.

**Table 2:** Numerical rating scale measurements of the studied groups

	Group E10 (*n* = 20)	Group E15 (*n* = 20)	Group E20 (*n* = 20)	*P* value
At PACU	0.5 (0–1)	0.5 (0–1)	0 (0–1)	0.936
1 h	1 (0–1)	0.5 (0–1)	0 (0–1)	0.821
2 h	1 (0–1)	1 (0–1)	0.5 (0–1)	0.324
4 h	1 (1–2)	1 (1–2)	1 (0–2)	0.611
6 h	2 (1–2.25)	2 (1–2)	1.5 (1–2)	0.292
8 h	3 (2–3)	2 (2–3)	2 (1–3)	0.210
12 h	3 (2.75–6)	3 (2–4.25)	2.5 (2–4)	0.212
18 h	2 (2–3)	2.5 (2–3)	2 (2–3)	0.812
24 h	3.5 (2–5)	3 (2–5)	3 (2–5)	0.933
36 h	4 (3–5)	3.5 (2–5)	3.5 (2–4)	0.364
48 h	3.5 (2–5)	4 (2.75–5)	3 (2–4)	0.512

Data is presented as median (IQR). *PACU* post-anaesthesia care unit

The mean ± SD of total postoperative morphine administration in the initial twenty-four h was 3.6 ± 1.23 mg within group E10, 3.3 ± 0.92 mg in group E15, and 3.3 ± 0.92 mg in group E20. The mean ± SD of total postoperative morphine administration in 1 st 48 h showed 6.8 ± 1.65 mg in group E10, 6.2 ± 1.81 mg in group E15, and 5.6 ± 1.76 mg in group E20. Time of first rescue analgesia, fentanyl administration during surgery, morphine administration following surgery within the initial first 24 h as well as 48 h, and ambulation time were insignificantly varied among all groups (*P* > 0.05). Table 3.

**Table 3:** Analgesia and ambulation time of the studied groups

	Group E10 (*n* = 20)	Group E15 (*n* = 20)	Group E20 (*n* = 20)	*P* value
Patients required intraoperative fentanyl	4 (20%)	2 (10%)	1 (5%)	0.322
Time of first rescue analgesia (h)	16.7 ± 5.69	17.3 ± 4.98	17.6 ± 5.26	0.862
Postoperative morphine consumption in 24th h (mg)	3.6 ± 1.23	3.3 ± 0.92	3.3 ± 0.92	0.575
Postoperative morphine consumption in 48th h (mg)	6.8 ± 1.65	6.2 ± 1.81	5.6 ± 1.76	0.103
Ambulation time (h)	2.2 ± 0.95	2.1 ± 0.97	2.1 ± 0.89	0.876

Data are presented as mean ± SD or frequency (%)

Patient satisfaction and complications (PONV, hypotension, and bradycardia) were insignificantly varied among all groups (*P* > 0.05). Table 4.

**Table 4:** Patient satisfaction and complications of studied groups

		Group E10 (*n* = 20)	Group E15 (*n* = 20)	Group E20 (*n* = 20)	*P* value
Patient Satisfaction	Extremely satisfied	16 (80%)	18 (90%)	18 (90%)	0.562
	Satisfied	4 (20%)	2 (10%)	2 (10%)	
Complications	Postoperative nausea and vomiting	3 (15%)	2 (10%)	2 (10%)	0.851
	Hypotension	3 (15%)	3 (15%)	4 (20%)	0.887
	Bradycardia	1 (5%)	1 (5%)	2 (10%)	0.765

Data are presented as frequency (%)

## Discussion

Our study evaluated different volumes of LA in ESPB during single-level lumbar spine fixation and found that the LVs (10 ml and 15 ml) groups had a non-inferior analgesic effect to the 20 ml group in terms of analgesia as shown in consumption of intraoperative fentanyl as well as postoperative morphine, pain score and time until first rescue analgesia. Hemodynamic measurements, ambulation time, patient satisfaction, and complications were comparable among the groups.

Lower LA volumes are important for several reasons, such as reducing the risk of systemic toxicity, tissue damage, inflammation at the injection site, and nerve injury. Moreover, using LVs in LA also has economic benefits [18, 19].

Supporting our conclusions, Abdella et al. [20] exhibited that a higher LA volume (40 ml) in ESPB in breast cancer surgeries does not improve the analgesic effect compared to the LVs (20 ml) in decreasing opioid consumption and postoperative pain.

Contrasted to our results, Zengin et al. [21] reported that the analgesic effects were notably improved using 30 ml of LA in ESPB compared to 20 ml in thoracotomy patients. This difference may be due to the different levels of LA injection (thoracic level compared to the lumbar level in our study), as well as different surgical techniques, which are possible explanations for the different results since the thoracotomy is more painful; they could require additional LA volume for ESPB on acute pain post thoracotomy. Moreover, the single-level fixation surgery in our study may require a lower LA volume.

The efficacy of ESPB relies on its diffusion inside compartments and the LA distribution to adjacent nerves. LA’s absorption and diffusion are essential for identifying ESPB quality, as it typically diffuses through inter-transverse connective tissue and distributes anteriorly to the ventral and dorsal rami of the spinal nerves, ultimately reaching the paravertebral space [22]. Cranially, the fascia extends from the nuchal fascia to the sacrum caudally. Due to ESP, LA agents spread across many levels, resulting in a wide-ranging and compelling block. LA diffusion can lead to differences in pain relief outcomes, and patients may experience variability in analgesia, leading to frustration or dissatisfaction [23].

The primary mechanism of analgesia from LA is their direct effect through physical dissemination and diffusion to neural structures in the fascial plane beneath the ES muscles, as evidenced by clinical, cadaveric, animal, and laboratory investigations. This assertion is supported by the injectate’s consistent involvement of the dorsal rami and its variable extension to the ventral rami of spinal nerves, with less frequent epidural spread [24, 25]. From 10 mL to 30 mL, there was a small but direct correlation between the volume of injectate with ESP and craniocaudal distribution (as determined by the number of spinal levels). The ventral surface of the ES muscle was intensely stained from L1 to L4 after 30 ml of injectate. No staining was observed in the epidural space, intervertebral foramina, psoas muscles, or lumbar plexus, and there was no profound dissemination of the transverse processes [26].

We did not include a control group in our trial as ESPB has proven to be effective in several trials in spine surgeries.

Choosing ESPB has shown to be effective in lumbar surgeries, a finding supported by multiple meta-analyses and reviews. Wilson et al. [27] reported significant reductions in cumulative pain scores within the first 48 h at rest and during activity with ESPB. Similarly, Fu et al. [28] found notably lower pain scores at rest and during movement within 48 h post-surgery in patients receiving ESPB, reinforcing the efficacy of this regional technique for postoperative analgesia regardless of the volume used.

The time to first rescue analgesia was similar across groups (approximately 17 h). This duration exceeds the average time reported by Muthu et al. [13], who found no substantial variations in the timing of the first analgesic request between ESPB and control. However, our findings contrast with Sun et al. [29], who reported that ESPB notably prolonged the time needed for the first analgesic request.

Regarding opioid consumption, it was low and comparable across groups. Duan et al. [30] demonstrated that ESPB notably reduced total opioid consumption. Similarly, Ma et al. [31] reported notably lower opioid use in the first 24 h post-surgery in patients receiving ESPB. The dose-dependent decline in intraoperative fentanyl requirement observed in our study mirrors the findings of Adhikari et al. [32], who reported notably reduced analgesic consumption (fentanyl) in ESPB.

Regarding patient satisfaction, most patients indicated they were extremely satisfied. Wilson et al. [27] reported improved patient satisfaction with ESPB. The comparable ambulation times across our volume groups (approximately 2.1 h) suggest that even the lowest volume (10 ml) provides sufficient analgesia to facilitate early mobilisation. While Muthu et al. [13] found no substantial variations in time to ambulation between ESPB and control groups, Oh et al. [33] reported shorter hospital stays in patients receiving ESPB, indicating that adequate analgesia may contribute to enhanced recovery parameters.

Our results indicate that similar efficacy of ESPB across groups facilitates early postoperative recovery, aligning with Adhikari et al. [32], who concluded that ESPB contributes to early postoperative recovery, reduced analgesic requirements, and shorter hospital stays compared to standard care. However, Duan et al. [30] found no significant differences between ESPB and control groups regarding surgery duration and hospital stay.

The incidence of PONV was low and comparable across all groups. Cui et al. [34] reported a notably reduced incidence of PONV with ESPB. Similarly, Ma et al. [31] demonstrated decreased rates of PONV with ESPB, and Sun et al. [29] observed a lower incidence of PONV associated with ESPB.

Hemodynamic complications such as hypotension and bradycardia showed no significant differences across groups, suggesting that increasing the volume of LA in ESPB does not adversely affect cardiovascular stability. This safety profile is critical in spine surgery patients, who may be vulnerable to hemodynamic fluctuations during the perioperative period. Notably, Fu et al. [28] reported no serious ESPB-related complications across included studies, supporting the safety of this regional technique at various volumes. Similarly, Ma et al. [31] found no ESPB-related complications in their meta-analysis, further reinforcing the favourable safety profile of this block.

One of our strengths is that we were the first to study the use of LVs of LA 10 and 15 ml in ESPB. Our trial was limited since it was single-centred, which affected the generalizability of results as differences in practice settings, patient demographics, or surgical techniques may affect the applicability of these results to other populations or institutions.

This study is limited by its relatively small sample size, single-centre design, the absence of a control group receiving systemic analgesia without ESPB, and a follow-up period restricted to 48 h, which may constrain the assessment of adverse effects and long-term outcomes.

Future research should comprise large-scale, multi-centre randomized controlled trials with appropriate control groups, varied doses, additives, anatomical levels, and extended follow-up periods to validate ESPB’s efficacy, safety, and long-term analgesic outcomes.

## Conclusions

Preoperative ESPB appears to be an effective analgesic technique for single-level lumbar spine fixation. LVs demonstrated non-inferiority to higher volumes regarding analgesic efficacy, hemodynamic stability, patient satisfaction, and complication rates. Additionally, the use of LVs may reduce the risk of LA toxicity and associated adverse effects.

## Data Availability

Data is available upon resonable request from corresponding author.

## Abbreviations

ASA: American Society of Anaesthesiology
BMI: Body Mass Index
ES: Erector Spinae
ESPB: Erector Spinae Plane Block
GA: General Anaesthesia
HR: Heart Rate
IQR: Interquartile Range
IV: Intravenous
LA: Local Anaesthetic
LVs: Lower Volumes
MAP: Mean Arterial Blood Pressure
NRS: Numerical Rating Scale
PONV: Postoperative Nausea and Vomiting
SD: Standard Deviation
USG: Ultrasound-Guided
